# Factorial Structure of the Morningness-Eveningness-Stability-Scale (MESSi) and Sex and Age Invariance

**DOI:** 10.3389/fpsyg.2019.00003

**Published:** 2019-01-17

**Authors:** Paula Vagos, Pedro F. S. Rodrigues, Josefa N. S. Pandeirada, Ali Kasaeian, Corina Weidenauer, Carlos F. Silva, Christoph Randler

**Affiliations:** ^1^INPP, Universidade Portucalense, Porto, Portugal; ^2^CINEICC, University of Coimbra, Coimbra, Portugal; ^3^CINTESIS, Department of Education and Psychology, University of Aveiro, Aveiro, Portugal; ^4^William James Research Center, University of Aveiro, Aveiro, Portugal; ^5^Department of Biology, Eberhard Karls University of Tübingen, Tübingen, Germany

**Keywords:** MESSi, three-factor structure, sex invariance, age-group invariance, distinctness, morning affect, eveningness, psychometric assessment

## Abstract

Assessing morningness-eveningness preferences (chronotype), an individual characteristic that is mirrored in daily mental and physiological fluctuations, is crucial given their overarching influence in a variety of domains. The current work aimed to investigate the best factor structure of an instrument recently presented to asses this characteristic: the Morningness-Eveningness-Stability-Scale improved (MESSi). For the first time, the originally proposed three-factor structure was pitched against a uni- and a two-factor solution. Another novelty was to establish that the best-fitting model would be invariant in relation to sex and age, two variables that influence chronotype. A Confirmatory Factor Analyses on the data obtained from a sample of 2096 German adults (age: 18–76; *M* = 25.5, *SD* = 7.64) revealed that the originally proposed three-factor structure of the MESSi – Morning Affect, Eveningness, and Distinctness – was the only one to achieve acceptable fit indicators. Furthermore, each scale obtained good internal consistency. In order to assess age invariance, following the literature on development and chronotype, our sample was divided into three age groups: 18–21 years, 22–31 years, and 32 years or older. Full measurement invariance of the three-factor model was found for sex and age. Regarding differences between sexes, females did not differ significantly from males in Morning Affect, but scored significantly lower on Eveningness and higher on Distinctness; this last result has been consistent across validation studies of the MESSi. With respect to age differences, the oldest group scored lower on Eveningness and Distinctness in comparison with the other two age-groups; the intermediate group (age: 22–31) scored lower on Morning Affect when compared to both the younger and older age groups. Additionally, both Eveningness and Distinctness were negatively correlated with age. This latter relation has been consistently reported in other validation studies. Our results reinforce the idea that the MESSi assesses three different components of chronotype in a reliable manner and that this instrument can be used to explore sex and age differences.

## Introduction

People differ in the time of the day in which the peak of mental and physiological functions occurs (chronotype) and can be classified in one of three types: morning-, evening-, or intermediate-types. Specifically, whereas in morning-types the peak of alertness arises in early hours, in evening-types it occurs in the afternoon/evening; the peak of intermediate-types is reached in the middle of the day (Schmidt et al., 2007; Adan et al., 2012). Concerning body temperature, the nadir occurs at 03:50 h in morning-types and at 06:01 h for evening-types (Baehr et al., 2000). This individual difference is relevant in a variety of domains. For example, it has been related to affective conditions (e.g., Randler et al., 2012; Oginska and Oginska-Bruchal, 2014), to health-related behaviors and problems (e.g., Fabbian et al., 2016; Suh et al., 2017), and to satisfaction with life (e.g., Randler, 2008; Jankowski, 2012). Chronotype also relates in different ways to various characteristics of personality (e.g., Lipnevich et al., 2017; Randler et al., 2017b). These examples justify the need to seriously consider this variable in research in an accurate manner (for a review, see also Adan et al., 2012).

Although chronotype can be assessed by different biological and objective methods (e.g., melatonin, body temperature and actimetry measurements), self-report questionnaires continue to be widely used (for a review, see Di Milia et al., 2013). Some examples are the Morningness-Eveningness Questionnaire (full form-MEQ, Horne and Östberg, 1976; reduced form-rMEQ, Adan and Almirall, 1991) or the Composite Scale of Morningness (CSM; Smith et al., 1989). More recently, Randler et al. (2016a) proposed another instrument to assess circadian preferences – the Morningness-Eveningness-Stability-Scale improved (MESSi) – that includes three subscales: Morning Affect, Eveningness, and Distinctness. Alike other instruments, the Morning Affect and Eveningness subscales indicate more morningness and eveningness preference, respectively. The Distinctness subscale measures the subjective amplitude or the range of fluctuations that occur during the day in the mental and physiological state of the individual. Whereas some individuals present a relatively stable state throughout the day (i.e., they do not feel strong differences in their state during the day), others experience larger variations (i.e., they perceive to be doing particularly well at some point in the day and worse in others); the first are considered to have a low amplitude and the later a high amplitude (Oginska, 2011; for related concepts, see also Folkard et al., 1979; Di Milia, 2005; Oginska et al., 2017).

The MESSi provides several improvements in relation to previous questionnaires (Di Milia et al., 2013; Randler et al., 2016a). For example, it includes a similar number of items formulated to assess morning and eveningness preferences, thus avoiding the morning-biased measurement characteristic of other instruments. It also clearly identifies the assessment of multiple dimensions. Even though previous instruments have been proposed to assess multi-dimensions of chronotype (e.g., Putilov, 1993; Roberts, 1998), and factor analysis exist on other morningness-eveningness scales (Neubauer, 1992; Brown, 1993; Caci et al., 2009), the MESSi suggests a novel three-factor structure. The wording of the items of the MESSi is also more updated and the questions are simpler to respond and interpret. Finally, the inclusion of the Distinctness, a dimension with growing recognized relevance in the assessment of circadian rhythm (Di Milia, 2005; Oginska, 2011; Dosseville et al., 2013), makes it a more complete instrument, which of course goes on charge of the length. Nevertheless, in comparison to other popular alternatives, the MESSi (composed of 15 items) adds a new dimension and still provides a shorter solution than the MEQ (composed of 19 items); as compared to the CSM (which contains 13 items) it only adds two items.

The MESSi has been submitted to several validation studies, namely in Germany, Spain, Iran, Portugal, and Slovenia (Randler et al., 2016a; Díaz-Morales and Randler, 2017; Diaz-Morales et al., 2017; Rahafar et al., 2017; Rodrigues et al., 2018; Tomažič and Randler, 2019). In short, all studies have replicated the three-factor internal structure (i.e., Morning Affect, Eveningness, and Distinctness) via exploratory (Randler et al., 2016a) or confirmatory factor analyses (Díaz-Morales and Randler, 2017; Diaz-Morales et al., 2017; Rahafar et al., 2017; Rodrigues et al., 2018). However, the factor structure has not been challenged by comparing a one-, two- or three-factor structure. These validation studies showed at least satisfactory internal consistency values (Cronbach’ alphas varying between 0.73 and 0.87 for Morning Affect, 0.80 and 0.84 for Eveningness, and 0.69 and 0.77 for Distinctness). Rahafar et al. (2017) further found the MESSi to be invariant at the configuration level only across the three countries involved in their study (Germany, Spain. and Iran); in other words, the three-factor model fitted acceptably for each country but the loadings and intercepts of items (particularly for the Eveningness measure) seem to differ across countries. Furthermore, Rodrigues et al. (2018) found evidence for strong invariance of the MESSi across men and women in a Portuguese sample of higher education students. Finally, though not explicitly testing for measurement invariance, Diaz-Morales et al. (2017) showed the three-factor model to acceptably fit different age groups (i.e., 17–30 years old and 31–65 years old). Therefore, testing factorial invariance is an important novel goal of this study.

Concurrent validity of the MESSi has also been confirmed against other typical questionnaires. Specifically, Morning Affect correlated positively and Eveningness correlated negatively with the CSM (Randler et al., 2016a) and with the rMEQ (Díaz-Morales and Randler, 2017; Faßl et al., 2018). Regarding Distinctness, the correlation between its scores and the CSM and the rMEQ was negative but lower than with the other two subscales (Randler et al., 2016a; Díaz-Morales and Randler, 2017). Moreover, in the study by Faßl et al. (2018), no correlations were found between Distinctness and the other subscales. Overall, these results suggest that Distinctness acts separately from Morning Affect and Eveningness. These authors also reported some preliminary evidence for the MESSi chronotype assessment using measures of actigraphy and of the sleep-wake rhythm.

The literature on circadian preferences has also explored how these change throughout the development and if there are differences between sexes. Studies that have assessed chronotype using the MESSi, have revealed inconsistent sex differences on Morning Affect and Eveningness (e.g., Díaz-Morales and Randler, 2017; Diaz-Morales et al., 2017; Rahafar et al., 2017). This inconsistency mimics that obtained when other instruments are used to asses chronotype and may be a result of (low) sample size and high variation in age (Randler, 2007; Adan et al., 2012). Regarding the subscale of Distinctness, the results have been very regular across all of the just mentioned studies, with females reporting higher Distinctness than males (e.g., Rahafar et al., 2017; Rodrigues et al., 2018).

The evaluation of chronotype in different age groups has revealed that children tend to be morning-oriented and then become more evening-oriented during adolescence (e.g., Roenneberg et al., 2004; Randler et al., 2017a). Morningness usually increases again, particularly after the age of 20/21 years, and tends to stabilize until individuals reach around the age of 30 (Roenneberg et al., 2004; Adan et al., 2012; Randler et al., 2016b). Some of the studies that have used the MESSi have reported positive relations between Morning Affect and age and negative relations between Distinctness and age (e.g., Díaz-Morales and Randler, 2017; Rodrigues et al., 2018). Regarding the relation between Eveningness and age, the results have been more irregular, with some reporting negative relations (e.g., Díaz-Morales and Randler, 2017; and some countries from the Rahafar et al., 2017 study) and others non-significant relations (Rodrigues et al., 2018).

Given the existing literature, the main aim of the current work was to test competing models for the factorial structure of the MESSi and the invariance across age classes and sex of the best fitting model. In other words, the current work aimed to test the originally proposed three-factor structure of the MESSi (Morning Affect, Eveningness, and Distinctness) against uni- and two-factor model solutions. The first comparison helps to establish the multidimensionality purpose that underlined the development of this instrument (Randler et al., 2016a). The second evaluation aims to explore the idea that morningness-eveningness corresponds to a single dimension (Di Milia and Randler, 2013; Diaz-Morales et al., 2017) that in turn differs from the dimension of Distinctness. Furthermore, we aimed to establish that the best-fitting model would be invariant in relation to sex and age. This is an important statistical procedure in psychometric research to assure comparability across the groups being considered (Schmitt and Ali, 2015). With the exception of the study by Rodrigues et al. (2018), no other validation study of the MESSi has directly investigated the invariance of its factorial structure concerning sex and no other study has looked at the invariance for age groups. Finally, we also explored the differences between sexes and among age groups in the scores of each subscale of the MESSi (Morning Affect, Eveningness, and Distinctness).

## Materials and Methods

### Sample

Participants were 2096 adults aged between 18 and 76 years (*M* = 25.5, *SD* = 7.64); two participants did not provide information on their age (0.1%). The majority of participants was female (*n* = 1458, 69.6% females; *n* = 619, 29.5% males); nineteen participants (0.9%) did not provide information on their sex. Men were significantly older than women (*M* = 26.51, *SD* = 8.65 and *M* = 25.03, *SD* = 7.06, respectively, *t*(980.79) = 3.76, *p* < 0.001). For data analysis purposes (see below), participants were divided into three age groups: 21 years old or younger (*n* = 693, 33%), 22–31 years old (*n* = 1127, 54%), and 32 years old or older (*n* = 276, 13%). Such division took into account some of the ages at which stronger changes in chronotype are expected to occur (c.f. Introduction) while also ensuring a reasonable number of participants per age group. Men and women were not evenly distributed by these age groups, χ^2^(2) = 9.04, *p* = 0.01, with men being overrepresented in the two younger groups and women being more prevalent in the older group, as compared to what was statistically expected.

### Instrument

The MESSi is a self-report instrument that includes 15 items from three other questionnaires. The original items are from the Composite Scale of Morningness (Smith et al., 1989), the Caen Chronotype Questionnaire (CCTQ, Dosseville et al., 2013) and the Circadian Energy Scale (CIRENS; Ottoni et al., 2011). The total of the items is divided in three subscales, each one composed of five items: Morning Affect, Eveningness, and Distinctness. The items related to the Morning Affect subscale measure morningness preferences (early schedules), whereas the items of the Eveningness subscale assess evening preferences (late schedules). The remaining five items constitute the Distinctness subscale, that is, the amplitude dimension of this instrument. Each item is responded using a 5-points Likert scale and scored with 1–5 points, although some of them are reverse coded. The previous validation studies mentioned in the Introduction have revealed good indexes, such as Cronbach’ alpha values for the three subscales ranging between 0.69 to 0.87.

### Procedure

#### Sampling and Data Collection

Data collection was done from 23.10.2017 until 13.11.2017. Students and employees of the Eberhard Karls University of Tübingen were contacted by e-mail and asked to participate in a study about sleep and sexual behavior. In that same e-mail they were informed that it was a short questionnaire study about chronotype and partnership and that it would last about 15 min. They were also told that an anonymized procedure was in place, that their data would be used only for research purposes, and that they could withdraw their participation at any time without any consequences. We also explicitly stated that it was a voluntary and unpaid study. Then, participants were directed to a website from “SoSci Survey” where they had to answer to the questions; the consent of the participants was implied by completing the questionnaire. The questions concerning the MESSi took approximately 5 min to complete. We did not control for double or triple access. Two participants were excluded from the sample due to being under 18 years of age.

#### Data Analyses

A Confirmatory Factor Analyses (CFA) approach was used to test for competing models that might underlie the internal structure of the MESSi. Three measurement models were tested: (1) a one-factor model including all 15 items; (2) a two-factor model considering a Morning Affect/Eveningness factor with 10 items and a Distinctness factor with 5 items; and (3) a three-factor model referring to a Morning Affect factor, an Eveningness factor, and a Distinctness factor, each with five items. For the two-factor model, the scoring of the items from the Eveningness scale were reversed turning them into items contributing to a Morningness evaluation as if we were dealing with a morning-eveningness continuum (rather than two separate subscales as initially intended). The fit of these models was judged based on the guidelines provided by Hair et al. (2014) for samples larger than 250 participants and instruments using between 12 and 30 items. Therefore, the models were considered to fit the data if showing comparative fit index (CFI) > 0.92 combined with standardized root mean square residual (SRMR) < 0.08 or with root mean square error of approximation (RMSEA) < 0.07. Only one of the tested models acceptably fitted the data (see results section) and so only its measurement invariance by sex and by age-groups was analyzed, based on a forward approach (Dimitrov, 2010). Firstly, configural invariance was established if the model was found to fit well within each group under analyses. Then, metric invariance was investigated, meaning that the model that constraints all loadings to be equal across groups should be as good a fit as the model posing no equality constraints on the groups (i.e., ΔCFI < -0.01; ΔSRMR < 0.03; ΔRMSEA < 0.03). Finally, scalar invariance was also tested, based on finding a non-expressive difference between the loading-constraint model and a model constraining all intercepts to be equal across groups (i.e., ΔCFI < -0.01; ΔSRMR < 0.03; ΔRMSEA < 0.01; Chen, 2007).

Following the establishment of measurement invariance, a latent mean comparison approach was taken for between and among group comparisons (i.e., sex and age-groups, respectively). These analyses were further complemented with effect sizes, descriptive data and a two between-factor ANOVA to control for the uneven distribution of men and women by age-groups. These last analyses, as well as the calculations of the Cronbach’s alpha as a measure for internal consistency, were carried out using the IBM SPSS Statistics 21. In turn, CFA, measurement invariance, latent mean comparisons, between factor correlation analyses and correlation analyses between subscales and age were ran using Mplus v7.4 (Muthén and Muthén, 2012).

## Results

Preliminary analysis showed the data on the 15 items of the MESSi for the 2096 participants were not multivariate normal (Mardia’s multivariate skewness statistic = 6.59, *p* < 0.001; Mardia’s multivariate kurtosis statistic = 281.42, *p* < 0.001; Korkmaz et al., 2014). Hence, and because there were no missing values, the Robust Maximum Likelihood estimator was used for confirmatory factor analyses and for measurement analyses. Also, non-parametric tests were used for the correlation analyses.

### Evidence Based on the Internal Structure of the MESSi

The three factor measurement model originally proposed for the MESSi (Randler et al., 2016a) was the only one to achieve acceptable fit indicators based on the combination between CFI and SRMR values; the one-factor and the two-factor solutions did not abide by the fit guidelines for any of the indices under consideration (c.f. Table 1). All three measures also achieved mostly good internal consistency values: α = 0.87 for Morning Affect, α = 0.85 for Eveningness, and α = 0.75 for Distinctness. Loading values were always significant and varied between 0.65 (CSM 4) and 0.84 (CCQ 4) for Morning Affect, between 0.44 (CCQ 11) and 0.91 (CCQ 2) for Eveningness, and between 0.46 (CCQ 6) and 0.72 (CCQ 15) for Distinctness (c.f. Supplementary Material). The Morning Affect scale correlated significantly (*p* < 0.001) and negatively with the Eveningness (*r* = -0.59) and the Distinctness (*r* = -0.38) scales; Eveningness and Distinctness were also positive and significantly correlated although at a borderline significance level and with a low correlation value (*r* = 0.06, *p* = 0.041).

**Table 1:** Confirmatory factor analyses on the internal structure of the MESSi.

				χ2	df	RMSEA	CI for RMSEA	CFI	SRMR
**Confirmatory factor analyses**									
	1 Factor measurement model			5539.60	90	0.170	0.166; 0.174	0.553	0.134
	2 Factor measurement model			4101.11	89	0.147	0.143; 0.151	0.671	0.096
	3 Factor measurement model			993.69	87	0.071	0.067; 0.74	0.926	0.053
		**Between-sex measurement invariance**							
			Female participants	747.03	87	0.072	0.067; 0.077	0.927	0.054
			Male participants	338.45	87	0.068	0.061; 0.076	0.919	0.053
		**Between-age-groups measurement invariance**							
			21 years old or younger	383.11	87	0.070	0.063; 0.077	0.917	0.055
			Between 22 and 31 years old	622.02	87	0.074	0.068; 0.079	0.920	0.058
			31 years old or older	191.77	87	0.066	0.053; 0.079	0.946	0.060

*df, degrees of freedom; RMSEA, root mean square error of approximation; CI, confidence interval; CFI, comparative fit index; SRMR, standardized root mean square residual. All chi-square values were significant at *p* < 0.001.*

Full measurement invariance by sex was established for the three-factor model given that it fitted well for female and male participants taken separately (i.e., configural invariance; c.f. Table 1)^1^, that forcing all item loadings to be equal between groups did not significantly worsened the fit of a non-constraint model (i.e., metric invariance; ΔCFI = 0.000, ΔRMSEA = -0.002 and ΔSRMR = 0.002), and, additionally, that forcing all item intercepts to be equal across groups again did not significantly worsened the fit of the loading constraint model (i.e., scalar invariance; ΔCFI = -0.004, ΔRMSEA = 0.000, and ΔSRMR = 0.003)^2^.

Evidence for the three levels of measurement invariance by age-groups was also found, namely configural invariance (c.f. Table 1)^3^, metric invariance (ΔCFI = 0.000, ΔRMSEA = -0.003, and ΔSRMR = 0.003), and scalar invariance (ΔCFI = -0.002, ΔRMSEA = -0.002, and ΔSRMR = 0.001)^4^.

### Between-Groups Comparisons

Latent mean comparisons indicate that women, compared to men, scored significantly lower on the Eveningness (latent mean = -0.029, *p* < 0.001) and significantly higher on the Distinctness scale (latent mean = 0.563, *p* < 0.001); scores on the Morning Affect scale did not differ significantly between sexes. The direction of these results reflect those found for the same measures and groups when taking the sum of the responses of the set of items composing each measure (c.f. Table 2, also for the descriptive measures found using the complete sample).

**Table 2:** Descriptive measures for the subscales of the MESSi and effect sizes for between-groups comparisons.

		Morning affect				Eveningness				Distinctness			
		
		M (SD)	25th	50th	75th	M (SD)	25th	50th	75th	M (SD)	25th	50th	75th
**Total sample**		15.54 (4.27)	13	16	19	15.83 (4.11)	13	16	19	17.06 (3.34)	15	17	19
**Sex**													
	Female	15.44 (4.29)	13	16	19	15.48 (4.2)	12	15	19	17.55 (3.19)	16	18	20
	Male	15.75 (4.19)	13	16	19	16.68 (3.76)	14	17	19	15.92 (3.41)	14	16	18
	Cohen’s *d*	0.07				0.30				0.49			
**Age-groups**													
	21 years old or younger	15.85 (3.94)	13	16	19	16.03 (3.94)	13	16	19	17.18 (3.28)	13	18	19
	Between 22 and 31 years old	15.19 (4.35)	12	15	19	15.89 (4.15)	13	16	19	17.19 (3.28)	15	18	19
	32 years old or older	16.18 (4.68)	13	16	20	15.11 (4.29)	12	15	18	16.21 (4.64)	14	16	19
	Partial eta-squared	0.008				0.005				0.010			

Concerning age, correlation analyses revealed that age correlated positively with Morning Affect (*r* = 0.08, *p* = 0.003) and negatively with Eveningness (*r* = -0.08, *p* < 0.001) and Distinctness (*r* = -0.125, *p* < 0.001). Furthermore, latent mean comparisons showed that the oldest group had the lowest scores on the Eveningness and Distinctness scales, compared to both the younger group (latent mean = -0.182, *p* = 0.012 and latent mean = -0.145, *p* = 0.038, respectively) and the group of participants aged 22–31 years old (latent mean = -0.269, *p* < 0.001 and latent mean = -0.281, *p* < 0.001, respectively). In turn, participants aged between 22 and 31 years had significantly lower scores on the Morning Affect when compared to the younger group (latent mean = -0.152, *p* = 0.002) and to the older group (latent mean = 0.217, *p* = 0.002). The direction of these results, again, is in line with that found for the same measures and groups when taking the sum of the responses of the set of items composing each scale (c.f. Table 2).

Because men and women were not evenly distributed by age-groups, we conducted an ANOVA including both age-groups and sex as between groups factors. Their interaction effect was non-significant for the Morning Affect [*F*(2,2076) = 2.308, *p* = 0.10], for the Eveningness, and for the Distinctness (both *F*s < 1). These results suggest that sex- and age-based differences on the MESSi seem to be independent of each other.

## Discussion

The MESSi provides new way of assessing circadian preferences while introducing several improvements as compared to other existing instruments. Here, we tested the originally proposed three-factor structure of the MESSi (Morning Affect, Eveningness, and Distinctness), against other possible factorial structures. Also, we assessed the factor invariance across age groups and sex. The current study addressed these novel issues using a large sample of participants. Our results confirmed that the originally proposed three-factor structure of the instrument provides a better fit to the data as compared to the alternatives of a one- and two-factor structure.

Some studies that have tested the concurrent validity of the MESSi against other instruments (e.g., MEQ) have found correlations of about the same size as ours (but of different direction) between both the Morning Affect and Eveningness (Diaz-Morales et al., 2017; Rodrigues et al., 2018); such results could suggest that morningness-eveningness is a unidimensional construct and not separate as proposed in the MESSi (Diaz-Morales et al., 2017). However, our results suggest that each of the three different factors contribute separately to the assessment of chronotype. Empirically, studies have further started to show that each of these dimensions relate in a differential and significant manner with health-related measures as well as with some personality characteristics (Diaz-Morales et al., 2017) which helps to establish the relevance of each of the three factors. Furthermore, each scale obtained good internal consistency (range 0.75–0.87) scores.

The correlations found among the subscales are in line with those reported in other studies. The correlations between Morning Affect and both Eveningness and Distinctness were negative and significant with a larger relation between the first two, as expected (Díaz-Morales and Randler, 2017; Rodrigues et al., 2018). The correlation between Distinctness and Eveningness was also significant but with a low positive correlation coefficient; a similar result was reported by Rodrigues et al. (2018) but others have revealed non-significant correlations (Diaz-Morales et al., 2017).

Establishing that the best-found model would be invariant for the variables of sex and age was also an important and novel goal of this work. Full measurement invariance of the three-factor model was obtained for these variables indicating that the MESSi can accurately reflect sex and age differences related to the constructs. Such results reassure researchers that the MESSi accurately grasps the constructs within sex- and age- diversified samples and is an appropriate instrument to compare the results between sexes and across age groups.

We also explored the differences between sexes and among age groups in the scores of each subscale of the MESSi. Even though our sample was composed of unequal groups per sex or age, the same results were obtained when using balanced-sized groups (see footnotes 2 and 4). The pattern of differences between sexes has been quite inconsistent across studies, particularly with respect to the dimensions of Morning Affect and Eveningness, but we were able to find some communality with our data. Specifically, our females scored lower than males on Eveningness and the difference was not significant for Morning Affect (Diaz-Morales et al., 2017, undergraduate sample; Rodrigues et al., 2018). On the other hand, the finding that females score higher on Distinctness than males has been more consistently reported (e.g., Rahafar et al., 2017).

Regarding age, our correlation results revealed that as participants get older, they tend to score lower on Eveningness and Distinctness and higher on Morning Affect. This last result is in agreement with the idea that after the end of adolescence, people tend to become more morning oriented (Roenneberg et al., 2004), a relation that has also been corroborated in other studies using the MESSi (Díaz-Morales and Randler, 2017; Rahafar et al., 2017; Rodrigues et al., 2018). On the other hand, the negative correlation between Eveningness and age has been replicated in some studies (e.g., Diaz-Morales et al., 2017) but not in others (Rodrigues et al., 2018; the correlation was negative but non-significant). The negative correlation between age and Distinctness obtained in our sample has also been found in most validation studies of the MESSi in which this relation was analyzed (e.g., Rahafar et al., 2017; Rodrigues et al., 2018). Note that the disparate results regarding the correlations between age and Morning Affect and Eveningness are in favor of the idea that the latter two are indeed different constructs. Finally, we found no significant interaction between age and sex, a result that differs from that reported by Diaz-Morales et al. (2017). As for the differences among the age groups, considering the scarceness of studies that have addressed them before, we refrain from discussing these data at this time.

The diversity of results regarding the relation between age and the three subscales of this instrument could be due to a number of factors such as the different age ranges that have been tested across studies and the differential sample sizes. Furthermore, there is a number of factors that seem to affect chronotype such as individual and environmental variables (e.g., age, sex and photoperiod at birth, longitude and altitude; Adan et al., 2012); consequently, one could expect variability across countries as these differ in many of these aspects. It is noteworthy, though, that some results have indeed been consistent such as finding that females score consistently higher on Distinctness than males and the negative correlation between age and Eveningness and Distinctness. Future studies should explore the factors likely underlying these consistencies and also those that might justify the discrepancies.

In sum, this study confirms that the best fitting model for our data include the three factors described in the original presentation of the MESSi: Morning Affect, Eveningness and Distinctness. We further demonstrated that such structure is invariant for the variables of sex and age which ensures researchers that all of the instrument can be reliably used to assess chronotype in males and females as well as in various age groups. We also provide additional information regarding the relation between these two variables and chronotype in our sample with contributes to a more global understanding of this variable across countries.

## Author Contributions

CR, AK, and CW designed the study and collected the data. PV, PFSR, JNSP, and CFS made the analyses and drafted the manuscript. All authors contributed to the writing and discussion and approved the manuscript.

## Conflict of Interest Statement

The authors declare that the research was conducted in the absence of any commercial or financial relationships that could be construed as a potential conflict of interest.

## Footnotes

1 Loading values for female participants varied between 0.45 (CCQ 6 and CCQ 11) and 0.92 (CCQ 2; c.f. Supplementary Material) and internal consistency values were 0.85 for Morning Affect, 0.86 for Eveningness and 0.75 for Distinctness. Loading values for male participants ranged from 0.39 (CCQ 11) to 0.89 (CCQ 2; c.f. Supplementary Material) and internal consistency values were 0.86 for Morning Affect, 0.81 for Eveningness and 0.73 for Distinctness.

2 The same results were attained when randomly selecting a subsample of 50% of the female sample (*n* = 702) to contrast with the complete male sample (*n* = 619). That proportion was chosen so that the male and female groups had a similar size. Further information on the results using this sample may be requested from the corresponding author.

3 Loading values for participants aged 21 years old or younger varied between 0.42 (CCQ 6) and 0.89 (CCQ 2; c.f. Supplementary Material) and internal consistency values were 0.85 for Morning Affect, 0.83 for Eveningness and 0.71 for Distinctness. Loading values for participants aged between 22 and 31 years ranged from 0.44 (CCQ 11) to 0.92 (CCQ 2; c.f. Supplementary Material) and internal consistency values were 0.88 for Morning Affect, 0.85 for Eveningness and 0.75 for Distinctness. As for the participants aged 32 years old or older, loading values were placed between 0.41 (CCQ 11) and 0.94 (CCQ 2; c.f. Supplementary Material) and internal consistency values were 0.89 for Morning Affect, 0.87 for Eveningness and 0.81 for Distinctness.

4 The same results were attained when randomly selecting a subsample of 33% of the participants aged 21 years old or younger (*n* = 232) and a subsample of 25% of the participants aged 22–31 years old (*n* = 305) to contrast with the complete sample of participants aged 32 years or older (*n* = 276). Those proportions were chosen to make group sizes as similar as possible. Further information on the results using this sample may be requested from the corresponding author.
