# Supplementary material for: Factorial Structure of the Morningness-Eveningness-Stability-Scale (MESSi) and Sex and Age Invariance
Source: Front Psychol. 2019 Jan 17;10:3. doi: 10.3389/fpsyg.2019.00003 (PMC6344426; doi:10.3389/fpsyg.2019.00003)
Supplement: Supplementary file 1 [file Table_1.pdf]

Supplementary Material

Loading values for the three-factor model of the MESSi, for the complete sample, by sex and by age groups

	Complete sample	Sex-based groups		Age-based groups		
		**Men**	**Women**	**21 years old or younger**	**Between 22 and 31 years old**	**32 years old or older**
**Morning Affect**						
CSM3 (…) getting up in the morning	.76	.73	.79	.74	.76	.77
CSM12 (…) recover your senses in the morning (…)	.83	.83	.84	.79	.84	.89
CSM4 (…) first hour after having awakened (…)	.65	.60	.66	.60	.66	.68
CIRENS 1 (…) energy level in the morning	.75	.73	.76	.75	.75	.79
CCQ4 (…) long time after awakening	.84	.82	.85	.81	.84	.87
**Eveningness**						
CIRENS 2 (…) energy level in the evening	.58	.49	.59	.56	.56	.65
CCQ5 (…) study in the evening	.79	.74	.82	.75	.81	.84
CSM13 (…) more an evening active person	.86	.83	.87	.85	.86	.88
CCQ2 (…) think the best in the evening	.91	.89	.92	.89	.92	.94
CCQ11 (…) excellent mood in the evening	.44	.39	.45	.45	.44	.41

	Complete sample	Sex-based groups		Age-based groups		
		Men	Women	21 years old or younger	Between 22 and 31 years old	32 years old or older
**Distinctness**						
CCQ6 (…) same throughout the day	.46	.40	.45	.42	.48	.46
CCQ8 (…) focus at any time of the day	.63	.60	.61	.56	.62	.77
CCQ10 (…) motivation is the same	.57	.51	.58	.55	.55	.64
CCQ14 (…) feel unable to do anything	.69	.68	.71	.70	.69	.73
CCQ15 (…) it is harder for me to think	.72	.73	.72	.73	.71	.79

Notes. Completely standardized loading values are presented. Only short versions of the items are presented; the full provisional English version of the items and the coding instructions are available at https://www.researchgate.net/publication/315011478_MESSi_English_ResGate_Items_and_Coding
